# Circulating micrornas as potential biomarkers of aerobic exercise capacity

**DOI:** 10.1152/ajpheart.00711.2013

**Published:** 2013-12-20

**Authors:** Frank C. Mooren, Janika Viereck, Karsten Krüger, Thomas Thum

**Affiliations:** ^1^Department of Sports Medicine, Institute of Sports Sciences, Justus-Liebig-University, Giessen, Germany; and; ^2^Institute of Molecular and Translational Therapeutic Strategies, Hannover Medical School, Hannover, Germany

**Keywords:** marathon run, maximum oxygen uptake, cardiovascular system, adaptation

## Abstract

Purpose microRNAs (miRs) are crucial intracellular mediators of various biological processes, also affecting the cardiovascular system. Recently, it has been shown that miRs circulate extracellularly in the bloodstream and that such circulating miRs change in response to physical activity. Therefore, the purpose of the current study was to investigate heart/muscle specific and inflammation related miRs in plasma of individuals before, directly after, and 24 h after a marathon run and to analyze their relation to conventional biochemical, cardiovascular, and performance indexes. Male endurance athletes (*n* =14) were recruited for the study after performing a battery of cardiac functional tests. Blood samples were collected before, directly after, and 24 h after a public marathon run. miR-1, miR-133, miR-206, miR-499, miR-208b, miR-21, and miR-155 were measured using individual Taqman assays and normalized to *Caenorhabditis elegans* miR-39 (cel-39) spike-in control. Moreover, soluble cardiac, inflammatory, and muscle damage markers were determined. As a result, skeletal- and heart muscle-specific miRs showed a significant increase after the marathon. The strongest increase was observed for miR-206. Twenty-four hours after the run, only miR-499 and miR-208b were returned to preexercise levels, whereas the others were still enhanced. In contrast, miR-21 and -155 were not affected by exercise. miR-1, -133a, and -206 correlated to aerobic performance parameters such as maximum oxygen uptake (V̇o_2max_) and running speed at individual anaerobic lactate threshold (*V*_IAS_). miR-1 showed a moderate negative correlation with fractional shortening, whereas miR-133a was positively related to the thickness of intraventricular septum. None of the miRs correlated with cardiac injury markers such as troponin T, troponin I, and pro-brain natriuretic peptide. In conclusion, these findings suggest a potential role for muscle- and heart-specific miRs in cardiovascular adaptation processes after endurance exercise. Moreover, the specific correlation of miR-1, -133a, and -206 to performance parameters indicated their potential role as biomarkers of aerobic capacity.

regular physical exercise improves aerobic power and is accompanied with an increase of survival in cardiovascular diseases (27). In contrast, low aerobic exercise capacity is a powerful predictor of premature morbidity and higher mortality from all causes, including cardiovascular diseases (6, 18). Therefore, regular dynamic endurance sessions are important parts of preventive exercise programs. Performance gains can be detected using different approaches of which measuring the maximum oxygen uptake (V̇o_2max_) is considered to be an important method. Thereby, V̇o_2max_ depends primarily on the components of oxygen delivery and the oxidative mechanisms of the exercising muscle. However, several recent reports have documented considerable interindividual variations of training responses among participants of longitudinal training studies despite standardized exercise stimuli (7). With V̇o_2max_ adaptations ranging from 0 to >100%, genetic factors have been proposed to be involved. Consequently, a heritability from improvements in aerobic exercise capacity in response to endurance training has been estimated to ∼45–50% (7, 8). Several approaches such as genome wide association studies, RNA expression profiling, and others have been used so far to characterize genetic biomarkers of aerobic exercise capacity, whereas gene regulatory mechanisms including microRNAs (miRs) have been rarely investigated (8, 22, 30).

miRs comprise a class of 19–25 nucleotides, short noncoding RNAs that modulate gene expression on the posttranscriptional level and play important roles in a wide range of physiological and pathological processes (1). Mature miRs are single stranded and derived from immature hairpin precursors. Combined with RNA-induced silencing complex and Argonaute proteins, miRs bind to the 3′-untranslated region (3′-UTR) of their target transcript, provoking translational repression or degradation of the mRNA (1, 5). Importantly, some miRs are expressed in a tissue-specific manner and can be related to specific biological functions. Whereas miR-1, -133, -206, and -499 are highly abundant in both the skeletal muscle and/or the heart, miR-208b is more specific to myocardial tissue (11, 34, 35). Other miRs such as miR-21 and -155 can be related to inflammatory processes (37). As intensive exercise is accompanied by a substantial inflammatory response, it is unsurprising that exhaustive exercise affects the profile of inflammatory miRs in peripheral blood mononucleated cells (29). Recently, it has been demonstrated that the levels of muscle-related miRs (also called “myomiRs”) are altered in response to both acute aerobic and resistance exercise, suggesting a regulatory role during muscle adaption (17, 28, 32). A role of miRs as alternative biomarkers of muscle damage has also been described (4). Moreover, some exercise-regulated miRs have been found to be involved in the expression of performance-related genes (22). This latter observation has raised the question whether miRs might be important molecular switches that determine exercise-related cellular and molecular adaptation and might function as predictors of future exercise performance.

miRs are also stable outside the cell and were identified as a new class of biomarkers. Incorporated into exosomes (24, 39, 41), microvesicles (12, 20), or protein complexes (2), miRs can therefore be detected in human plasma (25). Recent studies documented changes of such circulating miRs in response to physical activity. Exercise altered the plasma levels of miRs implicated in angiogenesis, skeletal, and cardiac muscle adaptation in an intensity-, type-, and duration-dependent manner (15, 40). Finally, the potential value of miRs as markers of a low-training status as well as future cardiovascular health was recently suggested (9).

Therefore, we hypothesized that the exercise-induced changes of selective skeletal/heart muscle-related miRs are related to athlete's aerobic exercise performance capacity and may thereby serve as fitness biomarkers. Furthermore, the aim of the study was to test whether the alterations of miRs correlate to conventional soluble cardiac, inflammatory, and muscle damage markers.

## MATERIALS AND METHODS

### 

#### Subjects and study design.

Fifteen male subjects were recruited for the study of which, finally, 14 were included. One athlete was excluded from analysis because he did not finish the marathon run. All subjects were endurance runners with normal dietary habits who did not take any medication. They performed regular endurance training, including intensive workouts. However, none of the subjects included into the study was involved in any exercise training for at least 3 days before the run. All subjects passed through a standardized procedure consisting of signing the consent statement, general medical checkup, echocardiography, and spiroergometry. The investigations were approved by the Ethics Committee of the University conformed to the 1975 Declaration of Helsinki.

#### Echocardiography.

A two-dimensional transthoracic echocardiogram was performed (Hewlett-Packard, Andover, MA). Dimensions of right ventricle, left ventricular end-diastolic and end-systolic diameters, interventricular septum thickness, and posterior wall thickness were measured according to the American Society of Echocardiography guidelines using bidimensional or M-mode images depending on the quality and angulation between the ultrasound beam and the left ventricle.

#### Spiroergometry.

During the test, subjects were tested for their V̇o_2max_ using a continuous, progressive exercise test on a treadmill ergometer (Ergo XELG90 Spezial, Woodway, Weil am Rhein, Germany). The initial velocity was 8 km/h, increasing every 3 min by 2 km/h. Respiration parameters were continuously analyzed using Quark b2 (Cosmed, Rome, Italy). Along with a respiratory quotient of 1.05 or higher, a maximal test was considered achieved when the oxygen uptake did not increase more than 2 ml·min^−1^·kg^−1^ at the highest level or before the participant left the treadmill. V̇o_2max_ was measured as liters of oxygen per minute (l/min) and subsequently calculated as V̇o_2max_ relative to body mass (ml·min^−1^·kg^−1^). Moreover, at the end of every performance level (8, 10, 12…km/h) until termination of the exercise test, blood samples were taken from the ear lobe for lactate measurements. Individual anaerobic lactate threshold was calculated according to the method of Dickhuth using the Ergonizer program (16).

#### Blood sampling.

Blood samples were taken after cannulation of the cubital vein. Resting blood samples were taken 2 days before the marathon because the athletes refused sampling initially before the marathon. However, resting blood sampling was performed in the morning at the same time to avoid diurnal bias. Additional samples were taken after and 24 h after the run.

#### RNA extraction.

Total RNA from plasma samples was prepared using miRNeasy Mini Kit (Qiagen-Sample & Assay Technologies, Hilden, Germany) according to the manufacturer's directions. A standard volume of each plasma sample (200 μl) was supplemented with 5 fmol *Caenorhabditis elegans* miR-39 (cel-miR-39; Sigma-Aldrich, St. Louis, MO) to normalize the results.

#### Quantification of miRs.

A panel of miRs was investigated, which have been recently related either to skeletal/heart muscle (miR-1, -133a, -206, -499, and -208b) or to fibrotic/inflammatory processes such as miR-21 and -155 (13, 37, 38). To quantify the abundance of mature miRs, standard quantitative (“real time”) polymerase chain reaction (qRT-PCR) was performed. For that, cDNA of the selected miRs was synthesized with miR-specific RT primers (TaqMan MicroRNA Reverse Transcription Kit; Applied Biosystems, Foster City, CA). qRT-PCR was carried out via TaqMan miR assays (Applied Biosystems) in a C1000 Thermal Cycler (Bio-Rad, Hercules, CA) according to the manufacturer's instructions. Each sample was run in duplicates. Cel-miR-39 levels were determined in parallel, using the same type of detection assay (Applied Biosystems) and microRNA expression was normalized to the cel-miR-39 expression.

#### Leukocyte counts/biochemical measurements.

Blood cell counts, hemoglobin, and hematocrit determinations were performed on plasma anticoagulated with ethylenediaminetetraacetate using a semiautomated hematology analyzer (F-820, Sysmex, Norderstedt, Germany). Creatine kinase (CK) and myocardial isoenzyme (CK-MB) as well as lactate dedydrogenase were measured spectrophotometrically according to the guidelines of the International Federation of Clinical Chemistry (Beckman Coulter, Krefeld, Germany). Amino-terminal pro-brain natriuretic peptide (pro-BNP) was analyzed by ElectroChemiLumineszenz ImmunoAssay (ECLIA; Roche Diagnostics, Mannheim, Germany). Troponin T and troponin I were measured by chemiluminescent microparticle immunoassay (Abbott Park, IL). Interleukin-6 and high-sensitive C-reactive protein (hsCRP) were measured by immunoturbidimetric assay (Beckman Coulter, Krefeld, Germany).

#### Statistics.

GraphPad Prism 5.01 and SPSS 18.0 packages were used. Variables were expressed as means ± SD and compared with the Kruskal-Wallis test. Pearson's correlation analysis was used to identify any significant relationships. In all cases, *P* < 0.05 was accepted as being significant.

## RESULTS

Anthropometric data and performance parameters of the long-distance runners are shown in Table 1. Values for right ventricle and fractional shortening were in the normal range, whereas dimensions of the left ventricle (left ventricular end-diastolic diameter, interventricular septum thickness, and posterior wall thickness) were around the upper level of the normal range as could be expected for runners with a mean training experience of about 7.6 ± 2.3 yr. Athletes performed the marathon distance in a mean time of 215 ± 30 min.

**Table 1.** General characteristics of marathon runners

Characteristics (*n* = 14)	Mean ± SD
Age, yr	42.8 ± 6.0
Body mass index, kg/m^2^	25.3 ± 2.3
Performance parameters	
V̇o_2max_, ml·min^−1^·kg^−1^	48.7 ± 9.6
Running speed at IAS, km/h	13.3 ± 1.1
Marathon time, min	215 ± 30
Echocardiographic data	
Right ventricle, mm	23.6 ± 2.1
Septum, mm	10.0 ± 1.2
LVDD, mm	59.2 ± 6.5
Posterior wall, mm	9.2 ± 2.0
FS, %	33.8 ± 7.0

Values are means ± SD. V̇o_2max_, maximum oxygen uptake; LVDD, left ventricular end-diastolic diameter; FS, fractional shortening.

The exercise stimulus resulted in alterations of leucocyte counts, cellular destruction parameters, cardiac markers, and inflammatory marker proteins (Table 2), some of which returned to preexercise levels 24 h later (lymphocytes/monocytes/Il-6), whereas CK and hsCRP continuously increased.

**Table 2.** Biochemical measurements

	Before	After	After 24 h
Leucocytes (10^3^/μl)	5.0 ± 0.8	13.4 ± 1.9^a,^^b^	6.1 ± 1.9^c^
Lymphocytes (10^3^/μl)	2.0 ± 0.4	1.4 ± 0.3^a,^^b^	2.0 ± 0.6
Monocytes (10^3^/μl)	0.5 ± 0.1	1.1 ± 0.3^a,^^b^	0.5 ± 0.2
Neutrophiles (10^3^/μl)	2.5 ± 0.5	10.9 ± 1.6^a,^^b^	3.7 ± 1.2^c^
Destruction parameters			
CK (U/l)	127 ± 53	419 ± 125^a,^^b^	598 ± 209^c^
LDH (U/l)	127 ± 20	208 ± 44^a,^^b^	153 ± 34^c^
Cardiac markers			
CK-MB (U/l)	10 ± 4	20 ± 8^a^	19 ± 8^c^
Troponin T (pg/ml)	2.2 ± 1.4	28.5 ± 16.9^a,^^b^	6.8 ± 5.4
Troponin I (ng/ml)	0.004 ± 0.004	0.068 ± 0.060^a,^^b^	0.020 ± 0.017
Pro-BNP (pg/ml)	15.5 ± 8.7	58.9 ± 30.4^a^	48.5 ± 32.8^c^
Inflammatory markers			
hsCRP (mg/l)	0.9 ± 0.6	1.5 ± 0.6^b^	10.8 ± 5.7^c^
IL-6 (pg/ml)	BD (set to 1)	8.7 ± 4.1^a,^^b^	BD (set to 1)

Values are means ± SD. CK, creatine kinase; LDH, lactate dehydrogenase; hsCRP, high-sensitive C-reactive protein; CK-MB, myocardial isoenzyme; pro-BNP, pro-brain natriuretic peptide; IL-6, interleukin-6; BD, below detection threshold.

a Significant difference between before/after the run;

b significant difference between after/24 h after the run;

c significant difference between before/24 h after the run.

**Fig. 1.**
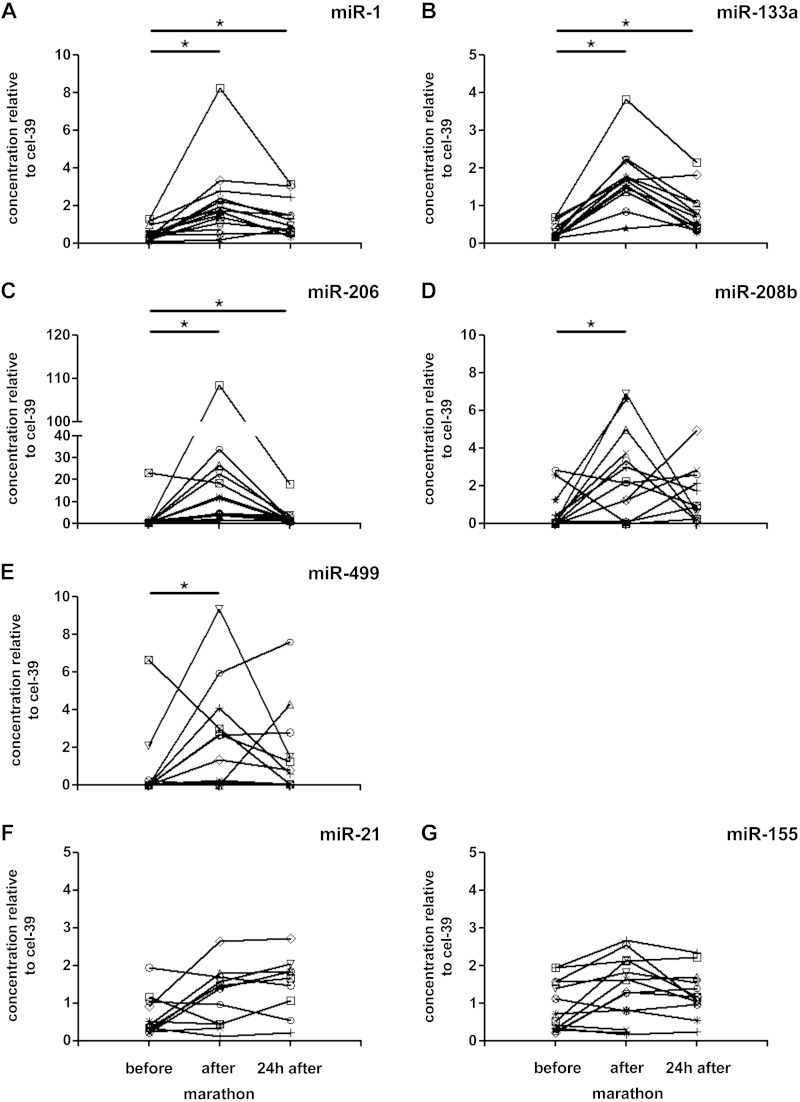
Effects of a marathon run on various circulatory microRNAs (miRs): miR-1 (*A*), miR-133a (*B*), miR-206 (*C*), miR-499 (*D*), miR-208b (*E*), miR-21 (*F*), and miR-155 (*G*). *P < 0.05, significance between the different time points. cel-39, *Caenorhabditis elegans* miR-39.

Immediately after the marathon, a significant increase of miR-1, -133a, -206, -208b, and -499 was observed (Fig. 1, *A–E*). The strongest increase was found for miR-206, which changed nearly 20-fold compared with preexercise levels. Twenty-four hours later, miR-499 and miR-208b were returned to preexercise levels, whereas the others were still enhanced significantly. In contrast, miR-21 (Fig. 1*F*) and miR-155 (Fig. 1*G*) were not affected by exercise. The increase of miR-1, -133a, and -206 was highly correlated among themselves (R values between 0.85–0.90; *P* ≤ 0.001), whereas no significant correlation was found in relation to or between the other miRs (R values between −0.17 and 0.49; *P* > 0.05). Only for the fibrosis/inflammatory miR-21 and -155, a significant correlation was detected (*R* = 0.78; *P* ≤ 0.01).

In a next step, we correlated the exercise-induced increase of circulatory miRs to indicators of performance capacity and cardiac function as well as markers of muscle damage and inflammation.

**Fig. 2.**
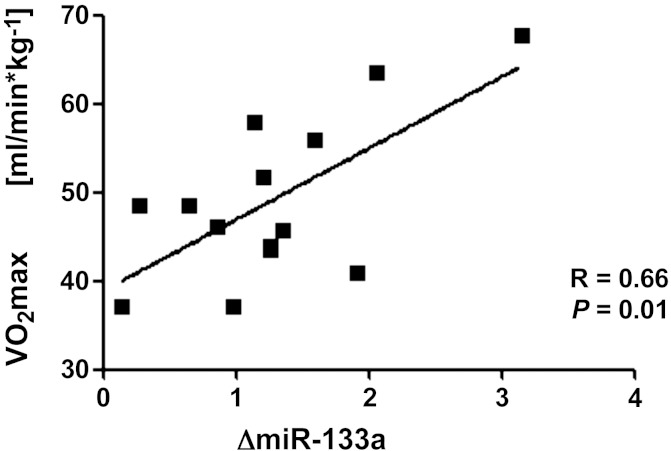
Exercise-induced increase of miR-133a correlated significantly with athlete's maximal oxygen uptake (V̇o_2max_).

For miR-1, -133a, and -206, moderate to strong correlations to V̇o_2max_ and running speed at individual anaerobic lactate threshold (*V*_IAS_) were found (Table 3, and Fig. 2). miR-1 showed a moderate, negative correlation with fractional shortening, whereas miR-133a was positively related to the thickness of intraventricular septum. In contrast, no correlations were found for miR-499, -208b, -21, and -155.

**Table 3.** Correlations of miRs to aerobic exercise capacity (as indicated by V̇o_2max_ and *V*_IAS_) and cardiac dimensions and function

	V̇o_2max_		*V*_IAS_		IVS		FS	
	*R*	*P*	*R*	*P*	*R*	*P*	*R*	*P*
ΔmiR-1	0.58	0.03	0.56	0.04	0.29	NS	−0.62	0.02
ΔmiR-133a	0.66	0.01	0.69	0.01	0.55	0.05	−0.45	NS
ΔmiR-206	0.71	0.004	0.67	0.01	0.43	NS	−0.35	NS
ΔmiR-208b	−0.25	NS	0.00	NS	0.31	NS	−0.13	NS
ΔmiR-499	−0.11	NS	0.46	NS	0.39	NS	−0.05	NS
ΔmiR-21	0.22	NS	−0.11	NS	0.15	NS	−0.50	NS
ΔmiR-155	−0.01	NS	−0.30	NS	0.09	NS	−0.41	NS

miR, microRNA; V̇o_2max_, maximal oxygen uptake; *V*_IAS_, running speed at individual anaerobic lactate threshold; IVS, interventricular septum; NS, not significant.

Finally, no correlations were found between miRs and cardiac biomarkers such as CK-MB, troponin T and I, and pro-BNP (data not shown). There were only discrete moderate correlations of single miRs to parameters of exercise-induced tissue destruction (CK and lactate dedydrogenase) and inflammatory markers (CRP and interleukin-6) (Table 4).

**Table 4.** Correlations between miRs and markers of inflammation and cell damage

	CRP		IL-6		LDH		CK	
	*R*	*P*	*R*	*P*	*R*	*P*	*R*	*P*
ΔmiR-1	−0.42	NS	−0.31	NS	−0.35	NS	0.31	NS
ΔmiR-133a	−0.33	NS	−0.53	0.04	−0.23	NS	0.61	0.02
ΔmiR-206	−0.53	0.04	−0.29	NS	−0.21	NS	0.46	NS
ΔmiR-208b	0.20	NS	−0.19	NS	0.01	NS	0.35	NS
ΔmiR-499	−0.06	NS	−0.08	NS	0.39	NS	0,49	NS
ΔmiR-21	0.36	NS	−0.24	NS	−0.16	NS	0.26	NS
ΔmiR-155	0.48	NS	−0.31	NS	−0.12	NS	0.26	NS

## DISCUSSION

An acute bout of endurance exercise induces an increase of heart and skeletal muscle-related circulating miRs. The course of myomiR-1, -133a, and -206 was highly correlated and slightly different from cardio-enriched miR-499 and -208b, suggesting a differential regulation during exercise. In contrast, the fibrosis/inflammation-related miR-21 and -155 showed no significant response to exercise. The most intriguing finding was, however, that the increases of the former three miRs correlated consistently to the athlete's aerobic performance capacity as indicated by V̇o_2max_ and *V*_IAS_. In contrast, their relation to skeletal and cardiac muscle destruction markers was rather limited, which might suggest alternative release mechanisms than just by cell death, thereby emphasizing their potential role as biomarkers of aerobic capacity.

The recent project was intended as a pilot study to test the suitability of circulating miRs as fitness biomarkers of exercise capacity, since miRs have been recently reported as indicators of various pathophysiological conditions such as myocardial infarction, heart failure, or various types of cancers (14, 25, 38). The sample size of the study was therefore limited to 14 participants. On the other hand, the study population was quite homogeneous with respect to sex (only male subjects), running experience (7.8 ± 2.4 yr), training history (61 ± 19 km/wk), and some performance data (Table 1). To maximize the accuracy of our approach, miR quantitative analysis was restricted to a subset of seven miRs, which have been described previously to be involved either in muscle tissue and cardiac development or inflammatory processes (10, 11). This contrasts to more comprehensive approaches using high-throughput screenings of all miRs, which would have enabled the analysis of specific clusters and/or regulatory patterns.

There are only a few studies available that have investigated the impact of exercise stimulus on miR expression either in tissues or in the circulation. The muscle and/or heart-specific miR-1, -133, -206, -208b, and -499 seem to be involved during skeletal and/or cardiac muscle development, which both are of particular importance for the improvements of V̇o_2max_ during exercise training programs (11, 13, 35). Specifically, our data suggest that the skeletal muscle-related miR-1, -133, and -206 seem to be more relevant for adaptation of aerobic capacity than the heart-related miR-208b and -499. Most of the studies so far have focused on the intracellular regulation of miR expression in various tissues such as muscle and immune cells (15, 17, 18, 29). During chronic exercise training, a downregulation of skeletal muscle miRs was observed independently whether endurance or strength training was applied (28). Similarly, in models of physiological cardiac hypertrophy, miR-1 and -133 were decreased (10). In contrast, an acute bout of exercise seems to be followed by a transient upregulation of at least miR-1 and -133a in skeletal muscle, which may result in a transient decrease of protein synthesis (28). Likewise, after endurance exercise, a depressed muscle protein synthesis has been described in the early postexercise period in a duration- and intensity-dependent fashion (31). The depleted cellular energy status seems both to impair adaptations of structural muscle proteins and to favor energy restoration and metabolic proteins.

Only three recent reports investigated the exercise-dependent regulation of circulatory miRs including miR-133a as in the current study. Whereas Baggish and coworkers (3) reported no changes of miR-133a, Uhlemann et al. (40) found an increase. In contrast, after resistance exercise Sawada and coworkers (33) found no alterations of miR-11a. These divergent results suggest most likely a differential regulation of miRs with respect to exercise type, duration, and intensity. Whereas the Baggish group (3) used a short exercise test, Uhlemann et al. (40) investigated the effects of a marathon run similar to the actual study. The postexercise increase of circulatory miRs may have different reasons. An apparent increase due to exercise-induced plasma contraction can be excluded as the expression levels were analyzed in relation to cel-miR-39. An upregulation of miRs by de novo miR transcription cannot be excluded under actual conditions. Another explanation might be the release of miRs from destroyed tissue.

Some studies have investigated the potential role of miRs as biomarkers of myocardial infarction or skeletal muscle damage, e.g., miR-1, -133, and -499 (4, 14, 38). Banzet et al. found that changes in circulatory miRs were dependent on the exercise mode. Only eccentric exercise known to induce a high degree of muscle damage was followed by increased plasma levels of miR-133a and -208b. However, two points make this explanation less likely for the current study. First, whereas miR-1, -133a, and -206 consistently correlate with V̇o_2max_, only miR-133a moderately correlated with serum CK levels, an indicator for skeletal muscle cell damage. Second, the time course of miRs and CK was completely different. It may, however, be possible that beside necrotic cells, programmed cell death may play a role as apoptotic bodies can contain large amounts of miR (42). Exercise is a well-known inducer of cell apoptosis, e.g., polymorphonuclear cells. We could demonstrate that lymphocyte apoptosis occurs in a similar time course like miR release (26).

Alternatively, it can be speculated that the exercise-induced increase of miRs results from intracellular processing of preexisting, premature miRs and their subsequent cellular secretion and excretion. A ceramide-dependent machinery for the secretion of miR-enriched exosomes has been recently described (21). But as the definitive underlying release mechanism were beyond the scope of our current study, future studies are necessary to address this question in more detail.

The consisting correlations of miR-1, -133a, and -206 to aerobic capacity raise the question about an epigenetic effect on the well-known variability of exercise performance after training interventions, resulting from complex gene-environment interactions. Several interventional studies reported considerable person-to-person variations of performance gains (7, 19, 23). For example, in the HEalth, RIsk factors, exercise Training And GEnetics (HERITAGE) Family Study, the average increase in V̇o_2max_ was ∼400 ml O_2_/min with a standard deviation of ∼200 ml/min (range, approximately −100 to approximately 1100 ml/min) (7). The heritability estimate of the V̇o_2max_ training response was about 50%, a level comparable with what has been observed in other family studies and in sets of identical and fraternal twins. This has stimulated the search for appropriate candidate genes which might help to predict gains in performance. In the latest update of the human gene map for physical performance and health-related phenotypes, 165 autosomal, 5× chromosomal, and 17 mitochondrial gene entries and quantitative gene loci were listed (30). Some of these have been recently characterized as genomic predictors of maximal aerobic capacity using different methodological approaches (8, 22, 36).

Therefore, we generated a cumulative list of these significant target genes (see supplementary Table 1 posted along with the online article) and searched whether they are addressed by heart und skeletal muscle-specific miRs. miRs from the heart such as miR-208b and -499 showed with one hit only limited relation to endurance-related genes. In contrast, a significant portion of the genes (about 15–20%) are modulated by either miR-1 or -206, thereby giving an additional, indirect evidence for the relevance of our findings.

Whereas most studies in this area have been performed with athletes, similar findings have also been reported for patients with chronic diseases such as heart failure, hypertension, etc. For example, the HERITAGE Family Study reported person-to-person variability for the blood pressure responses to exercise (mean decrease, 7 mmHg; range from +3 to −25 mmHg) (7). In clinical practice, about 25% of hypertensive patients can be classified as non- or low-responders regarding the blood pressure decreasing effects of regular exercise training. As low cardiorespiratory fitness is a powerful predictor of morbidity and cardiovascular mortality, there is a great need to understand how the exercise stimulus is turned into fitness (18). The investigation of the role of miRs seems to be a promising approach to improve our understanding about the underlying mechanisms as suggested also by a recent study from Bye et al. (9). They could demonstrate that resting plasma levels of miR-210, -222, and -21 were higher in healthy individuals with low V̇o_2max_ (9). Further longitudinal studies, however, are required to confirm these actual approaches. It seems worthwhile to include additional miRs, tissue biopsies, and exercise/training regimes to get more detailed and differentiated insights into this topic. But it can be expected to be helpful on our way into the era of individualized, optimized, and therefore more efficacious health recommendations and preventive training programs.

In summary, our data demonstrate the exercise-dependent regulation of heart and skeletal muscle-related circulating miRs. The potential suitability of selective miRs such as miR-1, -133a, and -206 as novel biomarkers of aerobic exercise capacity is suggested by their high correlation to standard performance parameters. The limited association between miRs and biochemical markers of cardiac function and skeletal muscle damage indicates their independence as biomarkers and might suggest specific cellular release mechanisms.

## GRANTS

This work was partly funded by the German Research Foundation Grant DFG-Th907/10-1.

## DISCLOSURES

All authors have completed the International Committee of Medical Journal Editors uniform disclosure form at http://www.icmje.org/coi_disclosure.pdf and declare the following: no support from any organization for the submitted work, no financial relationships with any organizations that might have an interest in the submitted work in the previous three years, and no other relationships or activities that could appear to have influenced the submitted work.

## AUTHOR CONTRIBUTIONS

F.C.M., K.K., and T.T. conception and design of research; F.C.M., J.V., and K.K. performed experiments; F.C.M., J.V., K.K., and T.T. analyzed data; F.C.M., J.V., K.K., and T.T. interpreted results of experiments; F.C.M., J.V., K.K., and T.T. prepared figures; F.C.M., J.V., and K.K. drafted manuscript; F.C.M., K.K., and T.T. edited and revised manuscript; F.C.M., J.V., K.K., and T.T. approved final version of manuscript.

## Supplementary Material

Table S1
