# Supplementary material for: Circulating micrornas as potential biomarkers of aerobic exercise capacity
Source: Am J Physiol Heart Circ Physiol. 2013 Dec 20;306(4):H557–63. doi: 10.1152/ajpheart.00711.2013 (PMC3920240; doi:10.1152/ajpheart.00711.2013)
Supplement: Table S1 [file tableS1.pdf]

Supplementary Table 1

High score prediction of microRNA binding sites within endurance-related target genes (based on references 7, 19, 32) as provided by at least one of the following data bases: (a) TargetScan; (b) PicTar; (c) miRanda; (d) MiRTarget2; (e) Diana microT v3.0

Gene Abbreviation	miR-1	miR-133a	miR-206	miR-208b	miR-499
219866_at	-	-	-	-	-
238619_at	-	-	-	-	-
244753_at	-	-	-	-	-
ACSL1	-	-	-	-	-
AMOTL2	-	-	-	-	-
AMPD1	-	-	-	-	-
BIRC7	-	-	-	-	-
BTAF1	**b)**	-	**b)**	-	-
BTNL9	-	**a)**	-	-	-
C9orf27	-	-	-	-	-
CAMTA1	-	-	-	-	-
CD44	-	-	-	-	-
CNTF	-	-	-	-	-
CPVL	-	-	-	-	-
CXCR5	-	-	-	-	-
DAAM1	**b), c)**	-	**b), c)**	-	-
DBX1	-	-	-	-	-
DEPDC6	-	-	**c)**	-	-
DIS3L	-	-	-	-	-
DNAJB1	-	-	-	-	-
ENOSF1	-	-	-	-	-
ENPP3	-	-	-	-	-

GRIN3A	-	-	-	-	-
H19	-	-	-	-	-
ID3	-	-	-	-	-
IER2	-	-	-	-	-
IL32	-	-	-	-	-
KCNH8	-	-	-	-	**c)**
KCNQ5	-	-	-	-	-
KLF4	**b)**	-	**b)**	-	-
LOC100130460	-	-	-	-	-
LOC100289626	-	-	-	-	-
LOC400950	**c)**	-	**c)**	-	-
MAST2	**e)**	-	**e)**	-	-
METTL3	-	-	-	-	-
MN1	-	-	-	-	-
NDN	-	-	-	-	-
NLGN1	-	-	-	-	-
NR3C1 (GR)	-	-	-	-	-
PAX3	**a), b), c), d)**	-	**a), b), c), d)**	-	-
PILRB	-	-	-	-	-
PRDM1	-	**e)**	-	-	-
QRSL1	-	-	-	-	-
RGS18	-	-	-	-	-
RTN4IP1	-	-	**d)**	-	-
RUNX1	-	-	-	-	-
SCN3A	-	-	-	-	-
SLC22A3	-	-	-	-	-
SMTNL2	-	-	-	-	-

SOX9	**c), e)**	-	**c), e)**	-	-
SYPL2	-	-	-	-	-
TET1	-	-	-	-	-
TTC6	-	-	-	-	-
UNKL	-	-	-	-	-
ZIC4	**b)**	-	**b)**	-	-
ZSWIM7	-	-	-	-	-
Total: 56	8	2	10	0	1
